# The Role of Mental Imagery in Depression: Negative Mental Imagery Induces Strong Implicit and Explicit Affect in Depression

**DOI:** 10.3389/fpsyt.2015.00094

**Published:** 2015-07-07

**Authors:** Stefanie Maria Görgen, Jutta Joormann, Wolfgang Hiller, Michael Witthöft

**Affiliations:** ^1^Department of Clinical Psychology and Psychotherapy, Johannes Gutenberg University of Mainz, Mainz, Germany; ^2^Department of Psychology, Yale University, New Haven, CT, USA; ^3^Department of Clinical Psychology, Psychotherapy, and Experimental Psychopathology, Johannes Gutenberg University of Mainz, Mainz, Germany

**Keywords:** depression, mental imagery, implicit affect, implicit measure, affect misattribution procedure

## Abstract

Mental imagery, seeing with the mind’s eyes, can induce stronger positive as well as negative affect compared to verbal processing. Given this emotion-amplifying effect, it appears likely that mental images play an important role in affective disorders. According to the subcomponents model of depression, depressed mood is maintained by both negative imagery (which amplifies negative mood) and less efficient positive imagery processes. Empirical research on the link between mental imagery and affect in clinical depression, however, is still sparse. This study aimed at testing the role of mental imagery in depression, using a modified version of the affect misattribution procedure (AMP) and the self-assessment manikin (SAM) to assess implicit (AMP) and explicit (SAM) affect elicited by mental images, pictures, and verbal processing in clinically depressed participants (*n* = 32) compared to healthy controls (*n* = 32). In individuals with a depressive disorder, compared to healthy controls, negative mental images induced stronger negative affect in the explicit as well as implicit measure. Negative mental imagery did not, however, elicit greater increases in explicitly and implicitly assessed negative affect compared to other processing modalities (verbal processing, pictures) in the depressed group. Additionally, a positive imagery deficit in depression was observed in the explicit measure. Interestingly, the two groups did not differ in implicitly assessed affect after positive imagery, indicating that depressed individuals might benefit from positive imagery on an implicit or automatic level. Overall, our findings suggest that mental imagery also plays an important role in depression and confirm the potential of novel treatment approaches for depression, such as the promotion of positive imagery.

## Introduction

Mental images are internal representations “giving rise to the experience of ‘seeing with the mind’s eye”’ without an appropriate sensory input [Ref. (1) p. 635]. Although we think about situations or problems using verbal representations (e.g., “I have to call a friend”) and/or mental images (e.g., “seeing the friend in our mind’s eye”), previous research and treatment approaches in the context of depression have focused almost exclusively on the verbal form of thinking (2, 3). Only recently has research begun to examine the impact of mental imagery in depression. Indeed, studies have found that intrusive memories and images are very common in depression and that these intrusions are related to strong emotions, such as sadness or fear, and high levels of distress (4–6). Additionally, the severity of depressive symptoms was found to be positively correlated with the vividness of deliberately generated negative mental images (7). Deeprose and Holmes (8) developed the impact of future events scale (IFES), a self-report measure for assessing the consequences during the previous week of intrusive images of future personal life events (e.g., “I had trouble concentrating”). Compared to control participants, high dysphoric as well as clinically depressed individuals reported a greater impact of involuntary negative images of the future (8, 9).

It seems that depression is not only associated with an increase in negative mental imagery but also with a decrease in positive mental imagery. In a non-clinical sample as well as in individuals with clinical depression, depression symptoms were associated with reduced vividness of images of positive future events (7, 9). Also, in individuals with higher depression scores compared to those with lower such scores, imagery of positively interpreted ambiguous words (e.g., “break”) elicited less pleasure (7).

Previous studies regarding the special link between imagery and emotion showed that compared to verbal processing, visualizing emotional scenarios elicits stronger positive as well as negative affective reactions [e.g., Ref. (10–12)]. Due to this powerful impact of imagery on emotional states, mental images might be of particular importance in affective disorders and might critically influence the affective characteristics of depression (2, 13). Based on these findings, Holmes et al. (2) have proposed a *subcomponents model of depression*, which emphasizes the role of mental imagery in the amplification and maintenance of depression. The model suggests that negative mental images can reinforce negative mood as well as a negative interpretation bias in the presence of ambiguity, which, in turn, can further worsen depressed feelings. Due to the high levels of negative affect in depression and the emotion-amplifying effect of mental imagery, mental images might be “particularly toxic” in depression [Ref. (2) p. 24]. Additionally, due to the mood-improving effect of positive imagery, deficits in positive mental images might result in lowered mood and thereby also maintain depression (2, 14). Systematic empirical research on these relations and especially investigations of clinical samples, however, are still sparse.

Furthermore, previous research has almost exclusively relied on explicit measures for assessing the emotional effects of imagery (15, 16). *Explicitly assessed affect* refers to intentional and controllable affective reactions and is usually measured with questionnaires or self-reports, in the context of mental imagery, for example, with the positive and negative affect schedule [PANAS; (17)]. Explicit measures, however, can be strongly affected by response biases and limited introspection (18). In particular, self-reports of emotional reactions can be influenced by self-concepts or beliefs about how specific events supposedly change emotional states (19, 20). Cognitions (verbal or visual) and emotional responses often arise very rapidly and automatically. Especially in depression, negative automatic cognitions and uncontrollable negative emotion are very common (3, 21). Theoretical models of depression suggest that depressive symptoms can emerge when automatically triggered implicit processes persist without a regulating influence of explicit processes (22–24). Indeed, previous studies have shown that implicit measures can be promising for the assessment of specific characteristics of depression. *Implicitly assessed affect* describes fast, automatic, and uncontrollable affective responses. Examples for implicit measures are the implicit association test [IAT; (25)], the extrinsic affective Simon task [EAST; (26)], and the affect misattribution procedure [AMP; (27)]. Haeffel et al. (23), for example, found that in contrast to the explicit measure, only the implicit measure for assessing vulnerability to depression (self-worth IAT) predicted affective responses to a subsequent distressing task. So, using implicit measures to investigate characteristics in depression might be a very promising addition to explicit measures. Previous work has examined the effects of imagery using objective measures, such as the impact of imagery on physiological reactions (28), neuronal activity (29), behavior (30), and performance tasks (31), but little is known about the impact of imagery on implicit affect.

The study by Ceschi et al. (15) represents a first attempt to use an implicit measure, the IAT, to assess implicit emotion (anxiety) induced by mental images. Anxious compared to calm or neutral mental images elicited higher levels of explicitly assessed anxiety. However, no effect was found for implicit anxiety. The authors proposed that the IAT measures implicit trait anxiety or the self-concept of anxiety, instead of affect changes after relatively short mental images (15, 16). Görgen et al. (16) therefore used an alternative implicit measure, a modified version of the AMP (27), to implicitly assess the emotional impact of mental images. In addition, this was the first study that investigates the relation between depressive symptoms and implicit affect changes induced by mental imagery. In this modified AMP (16), negative, neutral, and positive mental images were used as prime stimuli, followed by the presentation of an ambiguous stimulus (a Chinese character). The participants were asked to rate the pleasantness of the character on a four-point scale and were specifically instructed to avoid being affected by the prime stimuli when responding to the ambiguous stimuli. The principle behind this procedure is a misattribution effect (27, 32). Participants misattribute their affective reaction caused by the prime stimulus (e.g., a negative picture) to the presentation of the ambiguous stimulus. If the task is conducted as intended (not to intentionally rate the prime stimuli), the influence of the prime stimuli on the ratings of the ambiguous stimuli might be automatic and uncontrollable (27, 33). In this study by Görgen et al. (16), explicit affect was assessed with the self-assessment manikin (SAM ratings, valence dimension from unhappy to happy). The results showed that in the implicit measure mental imagery elicits more implicit negative affect than verbal processing. In the explicit measure, mental imagery compared to verbal processing showed an emotion-amplifying effect for both, negative and positive affect (experiment 1). The strong emotional impact of imagery is explained by the close link between sensory-based information and emotional processing circuits as well as by similarities of perception and imagery regarding their neuronal processing in the brain (13). In line with this explanation, the second experiment by Görgen et al. (16) showed that neutral and negative mental images are comparable to pictures in their implicit emotional impact. Compared to positive pictures, positive imagery induced even stronger implicit positive affect. The severity of depressive symptoms was negatively related to implicit affect after positive mental imagery. Importantly, implicit affect after positive imagery explained incremental variance in depressive symptoms beyond explicit affect (16).

To the best of our knowledge, no study has yet investigated the role of implicitly measured affect caused by mental images in clinical depression. The primary aim of the present study was therefore to investigate the emotion-amplifying effect of mental imagery in clinically depressed individuals, using an implicit (AMP) as well as an explicit measure of affect (SAM). By comparing the emotional impact of mental images, verbally processed stimuli, and “real” pictures, we can also determine the specific impact of mental images.

Testing the subcomponents model of depression (increased negative imagery with a particularly emotion-amplifying effect and decreased positive imagery), we postulated the following hypotheses:

1. *Negative mental imagery:* In depression, negative imagery elicits stronger negative affect than verbally processed negative stimuli, and comparable negative affect to that of pictures.
2. *Positive mental imagery:* In depression, positive mental images might have a comparable emotional impact to verbally processed positive stimuli and a significantly lower emotional impact than positive pictures.
3. *Compared to healthy controls:* In depression negative imagery might elicit greater increases in negative affect and positive imagery might improve the emotional state to a lesser extent.

The findings should be consistent in the explicit and implicit measure. Initial evidence suggests that the promotion of positive mental images can reduce depressive symptoms (14, 34, 35). An interpretation bias modification intervention using positive imagery was particularly successful in depressed patients who reported frequent everyday use of mental imagery [spontaneous use of imagery scale, SUIS; (34, 36)].

4. *The everyday use of mental imagery (SUIS)* should be positively correlated with positive affect after positive imagery in depression.

## Materials and Methods

### Participants

**Figure 1:**
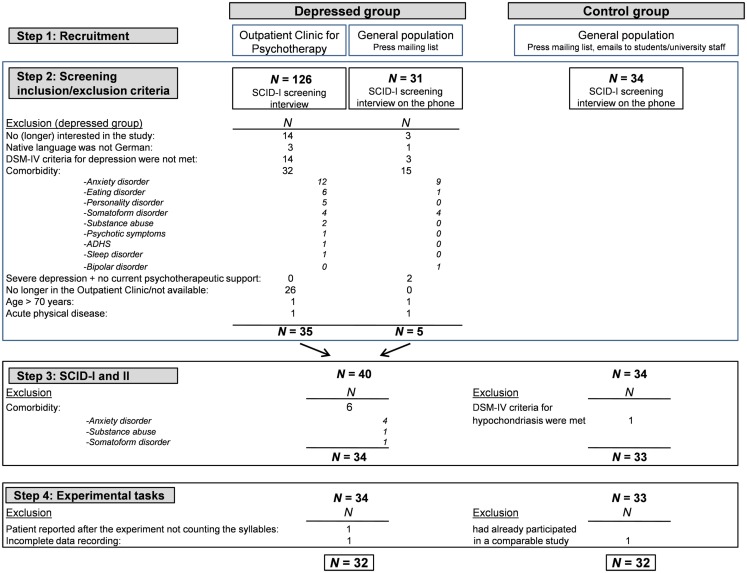
**Steps of the recruitment and selection process for the depressed and control groups**.

Participants were recruited from the Outpatient Clinic for Psychotherapy at the Johannes Gutenberg University of Mainz, via press mailing lists, and emails sent to all students and faculty of the university (see Figure 1). People who responded to the advertisement or email were first screened over the phone to determine whether they met basic inclusion and exclusion criteria. The telephone interview included questions of the structured clinical interview for DSM-IV axis I [SCID-I; (37)] to determine whether the person might be eligible for the depressed or control group. Potential participants were invited to the laboratory for a more extensive interview.

The recruitment and selection procedures as well as the inclusion and exclusion criteria for the depressed group are summarized in Figure 1. The main inclusion criterion for the depressed group was a current diagnosis of a depressive disorder (major depression and/or dysthymia) according to the diagnostic and statistical manual of mental disorders [DSM-IV; (38)]. Important exclusion criteria were comorbidity of any current other axis I or II disorder and a current suicidal tendency (for further exclusion criteria, see Figure 1).

After the telephone screening, only 16% (*n* = 5) of all volunteers who reported depressive symptoms were invited to the SCID. Most of the volunteers (*n* = 15) were excluded, because they suffered from an additional mental disorder (e.g., suspected anxiety disorder; see Figure 1). For ethical reasons, we excluded people with current severe depression who were not receiving psychotherapeutic support at the time of the telephone screening (*n* = 2).

After the screening phase, a total of 40 depressed individuals were invited to participate in the laboratory session. In these individual sessions, all diagnoses were first assessed by a trained and experienced clinical rater using SCID-I and -II (37). Eight of the 40 depressed individuals were further excluded from the study. According to the SCID, six participants were found to have at least one comorbid disorder (e.g., alcohol abuse, panic disorder, or pain disorder) and two participants were excluded for other reasons (problems with instructions and incomplete data recording; see Figure 1). Finally, the depressed group consisted of 32 participants with the following diagnoses: 22 participants with recurrent depressive episodes, 5 with a single depressive episode, 1 with a chronic major depression, 3 with dysthymia, and 1 with a double depression (combination of dysthymia and major depression). The inter-rater reliability (Cohen’s κ; first author and therapist; *n* = 29) for the SCID-I was κ = 1 for depressive disorder vs. no depressive disorder and κ = 0.65 for the type of depressive disorder. Six of the 32 depressed individuals had a history of another mental disorder (2 with substance abuse, 1 with alcohol dependency with more than 6 years complete remission, 1 with panic disorder with/without agoraphobia, and 1 with bulimia nervosa). At the time of the SCID, 31% of the depressed people reported taking antidepressants.

Healthy control participants consisted of individuals who had no current mental disorder and had never met criteria for a depressive disorder. In the control group, two participants were excluded: one person had already participated in a very similar study (possible practice effect or expectancies about the emotional impact of the imagery vs. verbal condition) and one met the DSM-IV criteria for alcohol abuse and hypochondriasis. Finally, the control group consisted of 32 never-depressed individuals (κ = 1, *n* = 24).

The study protocol obtained approval from the ethics committee of the Psychological Institute of the Johannes Gutenberg University of Mainz. All participants provided written informed consent and received monetary compensation for their participation ($40).

### Implicit measure of affect: Adapted affect misattribution procedure

The AMP (27) was modified and three different versions were developed: mental images, words, and “real” pictures were used as prime stimuli. First, participants received instructions to generate mental images (imagery condition) of the acoustically presented words, to count the syllables of the words (verbal condition), or to look at pictures presented in the center of the screen (picture condition). In the imagery instruction, we defined mental imagery as follows: “objects or scenes are imagined (by seeing, hearing, feeling …) in the absence of an actual perception.” It was emphasized that the generated mental images should be as concrete and vivid as possible. Based on the study by Görgen et al. (16), which demonstrated reliable results with prime durations of 3 s, participants had 3 s to generate and maintain mental images, to count syllables, or to look at the presented pictures. Following the presentation of the prime stimulus, a Chinese pictograph [randomly selected out of a set of 200 pictographs from Ref. (27)] was presented in the center of the screen for 200 ms. Subsequently, a mask of black and white noise was displayed until the participant rated the ambiguous stimulus on a four-point scale (33): “unpleasant [1],” “rather unpleasant [2],” “rather pleasant [3],” “pleasant [4].” In the preceding instructions, the participants were explicitly asked to avert possible impact of the acoustically presented stimuli on their judgments of the Chinese pictographs. Each version of the AMP (imagery, counting syllables, and pictures) comprised three practice trials with neutral stimuli and 60 test trials (10 negative, 10 neutral, and 10 positive words/pictures presented twice in random order).

### Explicit measure of affect: Self-assessment manikin

The SAM is a widely used non-verbal, pictorial measurement of affective responses (39). Following imagery, verbal processing, and pictures, participants were asked to rate their current emotional state on a five-point scale ranging from unhappy [1] to happy [5] (SAM valence dimension).

The five-point scale represents a relatively simple response format and was proved successful in a previously described study on mental imagery (15). Otherwise, the procedure was identical to the adapted AMP (including presentation times). As in the implicit task, each version of the explicit measure (imagery, counting syllables, and pictures) included three practice trials with neutral stimuli and 60 test trials (10 negative, 10 neutral, and 10 positive words/pictures presented twice in random order).

### Stimuli

We created 12 lists of words and 6 lists of pictures. In both the imagery and the verbal conditions, we used two word lists (implicit and explicit measure) of each valence (negative, neutral, and positive), resulting in 12 word lists (see Supplementary Material). The picture condition included two lists (implicit and explicit measure) of each valence (negative, neutral, and positive) (see Supplementary Material). The word stimuli were selected from the Berlin affective word list reloaded [BAWL-R; (40)], which is a database of about 3,000 German words with normative ratings on emotionality (valence), arousal, imageability, and information about other characteristics, such as frequency in the German language. Nouns with valence ratings below −1.3 were assigned to the negative category, between −0.8 and 0.8 to the neutral category, and above 1.3 to the positive category (seven-point scale ranging from −3 to +3) (41). To ensure that participants would be able to generate mental images, we selected words with imageability ratings ≥4 on a scale ranging from 1 (low imageability) to 7 (high imageability). All word lists were balanced in terms of imageability, word length, number of syllables, and word frequency, *F*s ≤ 0.25, *p*s ≥ 0.99 (Welch’s *F* if the homogeneity of variance was violated). The picture stimuli were drawn from the international affective picture system [IAPS; (42)]. To compare word and picture stimuli, we first standardized the emotionality and arousal values of the words and pictures. The six lists (imagery, verbal condition, and pictures, each in the explicit and implicit measure) of the same valence (see Supplementary Material) did not differ with regard to emotionality, *F*s ≤ 0.28, *p*s ≥ 0.92, and arousal, *F*s ≤ 0.35, *p*s ≥ 0.88.

### Self-report data

#### Depressive Symptoms

The severity of depressive symptoms was assessed with the *Beck depression inventory-II* [BDI-II; (43, 44)]. The 21 items based on the DSM-IV (38) are rated on a four-point scale ranging from 0 to 3. The German version has shown good reliability (α ≥ 0.84) and validity in clinical and non-clinical samples (45). In the present study, the Cronbach’s α was excellent (α = 0.95).

#### Measure of Mental Imagery

The *SUIS* is a self-report measure for assessing the everyday use of mental imagery (36). We used the extended German version with 17 items, which showed good reliability and validity (46). Items, such as “When I think about visiting a relative, I almost always have a clear mental picture of him or her,” are rated on a five-point scale ranging from 1 (never appropriate) to 5 (always completely appropriate). In the present study, the SUIS showed an internal consistency of α = 0.87.

#### Manipulation Check

To check compliance with the instructions in the experimental tasks, participants responded to the following questions on a five-point scale ranging from 1 (not at all) to 5 (very much): “How much were you able to (a) mentally imagine the presented words, (b) count the syllables of the words, (c) look at the presented pictures?” “How much were you able to concentrate on (a) mental images, (b) counting syllables, (c) looking at the pictures for the entire time interval?” “How difficult did you find (a) the mental imagery task, (b) counting syllables, (c) looking at pictures?” Participants were also asked: “How often did your mental images include autobiographical memories?”

### Procedure

Participants who responded to the advertisement or email took part in the telephone screening. The advertisement, entitled “Research on the role of mental imagery in depression,” was addressed to people who suffered from depressive symptoms, but had no other psychological problems. In the laboratory, all participants were tested individually. After giving written informed consent, SCID-I and -II were administered by trained psychologists. Following the SCID, each participant completed all experimental tasks: implicit affect after verbal processing, explicit affect after verbal processing, implicit affect after mental imagery, explicit affect after mental imagery, implicit affect after pictures, and explicit affect after pictures. Participants were randomly assigned to one of two orders either the verbal or the imagery condition was presented first. Due to the principle of implicit measures of concealing the intention of the measurement and of assessing more automatic and unintentional emotional reactions the influence of implicit measures on subsequent explicit measures might be smaller than vice versa (16). So, the implicit measure always preceded the explicit measure. After the experimental tasks, participants answered questions regarding socio-demographic data and manipulation check. Finally, they completed the BDI-II and the SUIS. A laboratory session lasted about 2.5 h.

### Statistical analyses

In the experimental tasks, we calculated means for each valence condition. To test hypotheses 1–3, we computed repeated-measures ANOVAs with group (depressed vs. control) as a between-subjects factor and condition (verbal processing vs. mental imagery vs. pictures) as well as valence (negative vs. neutral vs. positive) as within-subject factors. If the assumption of sphericity was violated, the Greenhouse–Geisser correction is reported. An alpha level of 0.05 (two-tailed) was adopted. Partial eta-squared ($\eta_{\text{p}}^{2}$) was calculated as effect size for ANOVA results and Cohen’s *d* as effect size for follow-up *t* tests. Hypothesis 4 was tested by a correlation analysis (Pearson test, *p* < 0.05).

## Results

### Participant characteristics and questionnaire data

Demographic characteristics and questionnaire data of the clinically depressed and control groups are presented in Table 1. The depressed and control groups did not differ with regard to sex, age, and education. As expected, depressed individuals reported significantly more depressive symptoms, *t*(62) = 12.28, *p* < 0.001, *d* = 3.07. The difference in the SUIS did not reach significance, *t*(62) = 1.67, *p* = 0.100, *d* = 0.42. Regarding the manipulation check, the two groups did not differ significantly in their ability to generate mental images (following the imagery instruction) and in the level of concentration on images and pictures. In contrast, depressed individuals rated themselves as less able to count syllables, *t*(62) = −2.73, *p* < 0.01, *d* = 0.68, and to look at the presented pictures, *t*(62) = −2.17, *p* < 0.05, *d* = 0.55, and reported lower levels of concentration on counting syllables, *t*(62) = −4.16, *p* < 0.001, *d* = 1.03. Furthermore, depressed participants rated all tasks as significantly more difficult, *t*(62) ≥ 2.15, *p*s ≤ 0.036, *d*s ≥ 0.54. In both groups, autobiographical events were often included in mental images, *t*(62) = 0.34, *p* = 0.734, *d* = 0.08.

**Table 1:** **Demographic characteristics and questionnaire data for the clinically depressed and control groups**.

Variables	DG (*N* = 32)	CG (*N* = 32)	Statistics (*df*)	Effect size
	*M* (SD)	*M* (SD)			
Female, *n* (%)	21 (66%)	24 (75%)	0.67 (1)	0.10
Age (years)	36.13 (12.68)	38.38 (12.32)	−0.72 (62)	0.18
Higher education, *n* (%)	23 (72%)	24 (75%)	0.08 (1)	0.04
BDI-II	20.66 (7.83)	2.09 (3.44)	12.28 (62)**	3.07
SUIS-17	53.91 (12.31)	48.81 (12.06)	1.67 (62)	0.42
**Manipulation check**				
Mental imagery				
Ability	4.12 (0.75)	4.22 (0.66)	−0.53 (62)	0.14
Concentration	3.50 (0.84)	3.72 (0.73)	−1.11 (62)	0.28
Difficulty	2.63 (1.01)	1.87 (0.98)	3.02 (62)**	0.76
Autobiographical	3.09 (1.20)	3.00 (0.98)	0.34 (62)	0.08
Counting syllables				
Ability	3.47 (1.11)	4.19 (1.00)	−2.73 (62)**	0.68
Concentration	2.66 (1.00)	3.78 (1.16)	−4.16 (62)**	1.03
Difficulty	2.47 (1.27)	1.81 (1.18)	2.15 (62)*	0.54
Looking at pictures				
Ability	3.91 (0.89)	4.38 (0.83)	−2.17 (62)*	0.55
Concentration	3.66 (1.34)	3.44 (1.13)	0.71 (62)	0.18
Difficulty	3.19 (1.12)	2.44 (1.19)	2.60 (62)*	0.65

*DG, depressed group; CG, control group; *M*, mean; SD, standard deviation; BDI-II, Beck depression inventory-II; SUIS-17, The German 17-item version of the spontaneous use of imagery scale*.

*For dichotomous variables, we calculated the chi-squared test with *w* as the effect size estimation (47). For continuous variables, the results of sample *t* tests with Cohen’s *d* as effect size are reported*.

*p < 0.05.

**p < 0.01.

### Explicit affect (SAM)

The internal consistencies for the SAM ratings were excellent, ranging from α = 0.91 (positive pictures) to α = 0.98 (negative pictures and counting syllables of negative words). A 2 (group: depressed vs. control) × 3 (condition: mental imagery vs. verbal processing vs. pictures) × 3 (valence: negative vs. neutral vs. positive) repeated-measures ANOVA showed significant main effects of group, *F*(1, 62) = 29.89, *p* < 0.001, $\eta_{\text{p}}^{2}=0.33$ (DG < CG), of condition, *F*(2, 124) = 3.48, *p* < 0.05, $\eta_{\text{p}}^{2}=0.05$ (pictures < verbal), and of valence, *F*(1.09, 67.30) = 93.30, *p* < 0.001, $\eta_{\text{p}}^{2}=0.60$ (neg < neu < pos). Additionally, we found significant interactions of group by valence, *F*(2, 124) = 6.40, *p* < 0.01, $\eta_{\text{p}}^{2}=0.09,$ and condition by valence, *F*(2.42, 149.81) = 16.87, *p* < 0.001, $\eta_{\text{p}}^{2}=0.21.$ These effects, however, were qualified by a three-way group by condition by valence interaction, *F*(4, 248) = 3.33, *p* < 0.05, $\eta_{\text{p}}^{2}=0.05.$

**Figure 2:**
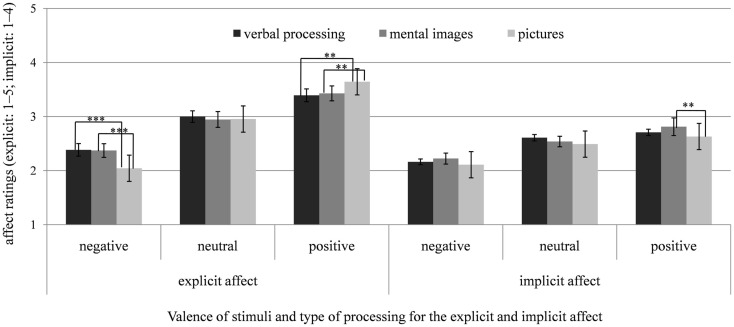
**Means and SEs of the explicit (SAM, scale: 1–5) and implicit affect ratings (AMP, scale: 1–4) after verbal processing (counting syllables), mental imagery, and looking at pictures of negative, neutral, and positive stimuli for the depressed group (DG)**. *p < 0.05; **p < 0.01; ***p < 0.001.

To further test the hypotheses (i.e., the emotion-amplifying effect of mental imagery in depression) and to clarify this three-way interaction, we computed separate 3 (condition) × 3 (valence) ANOVAs for each group. In the *depressed group*, the ANOVA showed a significant interaction of condition by valence, *F*(2.89, 89.57) = 14.37, *p* < 0.001, $\eta_{\text{p}}^{2}=0.32.$ In contrast to hypothesis 1, mental imagery and verbal processing of negative stimuli did not differ significantly regarding their emotional impact, *t*(31) = 0.15, *p* = 0.882, *d* = 0.02, and negative pictures elicited significantly greater increases in negative affect than negative imagery, *t*(31) = 4.39, *p* < 0.001, *d* = 0.52. Consistent with hypothesis 2, we found no significant difference in the emotional responses to positive imagery and verbally processed positive stimuli, *t*(31) = 0.50, *p* = 0.623, *d* = 0.06, and positive pictures elicited stronger positive affect than positive mental images, *t*(31) = 3.25, *p* < 0.01, *d* = 0.38. Comparing pictures and verbal processing, negative and positive pictures elicited more negative and positive affect than verbally processed negative and positive stimuli, *t*s(31) ≥ 3.83, *p*s ≤ 0.01, *d*s ≥ 0.47. For neutral stimuli, there were no significant differences between the conditions, *t*s(31) ≤ 1.24, *p*s ≥ 0.23, *d*s ≤ 0.17. Means of the affective ratings after verbal processing, mental imagery and looking at pictures for the depressed group are illustrated in Figure 2.

**Figure 3:**
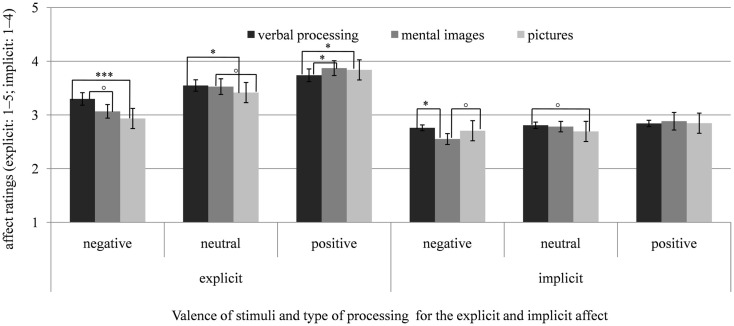
**Means and SEs of the explicit (SAM, scale: 1–5) and implicit affect ratings (AMP, scale: 1–4) after verbal processing (counting syllables), mental imagery, and looking at pictures of negative, neutral, and positive stimuli for the control group (CG)**. ^∘^*p* < 0.10; *p < 0.05; **p < 0.01; ***p < 0.001.

**Figure 4:**
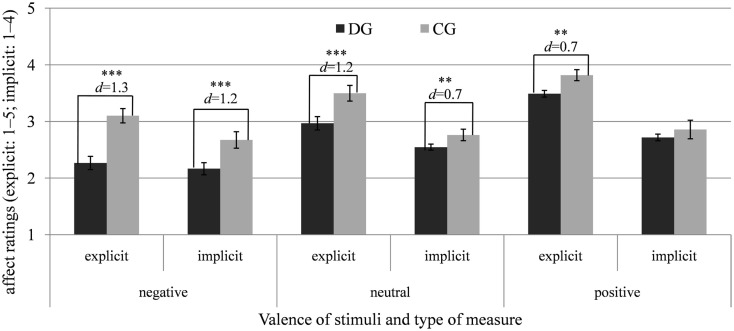
**Means and SEs of the explicit (SAM, scale: 1–5) and implicit affect ratings (AMP, scale: 1–4) after negative, neutral, and positive stimuli (pooled for the conditions) for the depressed (DG) and control group (CG)**. *p < 0.05; **p < 0.01; ***p < 0.001; *d* = Cohen’s *d* (effect size).

In the *control group*, we also found a significant interaction of condition by valence, *F*(1.90, 58.95) = 6.54, *p* < 0.01, $\eta_{\text{p}}^{2}=0.17.$ In line with the emotion-amplifying effect of mental imagery, positive mental images elicited stronger positive affect than positive stimuli in the verbal condition, *t*(31) = 2.22, *p* < 0.05, *d* = 0.25, and negative imagery elicited marginally more negative affect than negative stimuli in the verbal condition, *t*(31) = 1.94, *p* = 0.061, *d* = 0.30. Negative as well as positive mental images and pictures did not differ in their emotional impact, *t*s(31) ≤ 1.09, *p*s ≥ 0.284, *d*s ≤ 0.17. Additionally, negative pictures induced more negative affect than verbally processed negative stimuli, *t*(31) = 4.56, *p* < 0.001, *d* = 0.49, and positive pictures more positive affect than verbally processed positive stimuli, *t*(31) = 2.08, *p* < 0.05, *d* = 0.20. Regarding neutral stimuli, the neutral pictures elicited more negative affect than the verbal processing of neutral stimuli, *t*(31) = 2.36, *p* < 0.05, *d* = 0.23, and marginally more negative affect than neutral mental images, *t*(31) = 1.88, *p* = 0.07, *d* = 0.20. Figure 3 illustrates the means of affective ratings after verbal processing, mental imagery, and looking at pictures for the control group and Figure 4 shows the comparison of both groups regarding negative, neutral, and positive stimuli pooled for the conditions.

The means (see also Figure 4) suggest that the effects between the depressed and control group might be stronger for negative than for positive stimuli (interaction of group by valence). So, we additionally considered separate ANOVAs for each condition to clarify the three-way interaction. In the mental imagery condition, there were only significant main effects of group, *F*(1, 62) = 25.63, *p* < 0.001, $\eta_{\text{p}}^{2}=0.29$ (DG < CG), and valence, *F*(1.13, 69.86) = 64.94, *p* < 0.001, $\eta_{\text{p}}^{2}=0.51$ (neg < neu < pos). Regarding the verbal condition, the main effects of group, *F*(1, 62) = 28.87, *p* < 0.001, $\eta_{\text{p}}^{2}=0.32$ (DG < CG), and valence, *F*(1.15, 71.47) = 46.39, *p* < 0.001, $\eta_{\text{p}}^{2}=0.43$ (neg < neu < pos), as well as the interaction of group by valence were significant, *F*(2, 124) = 7.24, *p* < 0.01, $\eta_{\text{p}}^{2}=0.11.$ The effect sizes for the difference between both groups were larger for negative (*p* < 0.001, *d* = 1.33) and neutral (*p* < 0.001, *d* = 1.19) than for positive stimuli (*p* < 0.05, *d* = 0.65). In the picture condition, there were also significant main effects of group, *F*(1, 62) = 23.47, *p* < 0.001, $\eta_{\text{p}}^{2}=0.28$ (DG < CG), and valence, *F*(1.17, 72.20) = 113.00, *p* < 0.001, $\eta_{\text{p}}^{2}=0.65$ (neg < neu < pos), as well as a significant interaction of group by valence, *F*(2, 124) = 8.86, *p* < 0.001, $\eta_{\text{p}}^{2}=0.13.$ Similar to the verbal condition, affective reactions to negative (*p* < 0.001, *d* = 1.23) and neutral stimuli (*p* < 0.001, *d* = 0.97) were more negative in depression, whereas the two groups did not differ regarding positive stimuli (*p* = 0.11, *d* = 0.40).

In accordance with hypothesis 3, in the depressed compared to the control group, negative imagery elicited significantly lower affect, *t*(62) = 3.85, *p* < 0.001, *d* = 0.96, and emotional responses to positive imagery were significantly reduced, *t*(62) = 3.15, *p* < 0.01, *d* = 0.79.

### Implicit affect (AMP)

The internal consistencies of the AMP were all acceptable to good, ranging from α = 0.73 (counting syllables of neutral words) to α = 0.92 (negative pictures). For implicit affect, the 2 (group) × 3 (condition) × 3 (valence) repeated-measures ANOVA also revealed a significant main effect of group, *F*(1, 62) = 16.65, *p* < 0.001, $\eta_{\text{p}}^{2}=0.21$ (DG < CG), a marginally significant effect of condition, *F*(1.80, 111.32) = 3.13, *p* = 0.053, $\eta_{\text{p}}^{2}=0.05$ (pictures < verbal), and a significant effect of valence, *F*(1.17, 72.58) = 21.83, *p* < 0.001, $\eta_{\text{p}}^{2}=0.26$ (neg < neu < pos).

These effects were qualified by a significant interaction of group by valence, *F*(2, 124) = 5.93, *p* < 0.01, $\eta_{\text{p}}^{2}=0.09.$ The interactions of group by condition, *F*(2, 124) = 2.58, *p* = 0.080, $\eta_{\text{p}}^{2}=0.04,$ condition by valence,*F*(3.25, 201.58) = 1.68, *p* = 0.156, $\eta_{\text{p}}^{2}=0.03,$ and the three-way interaction of condition by valence by group, *F*(4, 248) = 1.78, *p* = 0.133, $\eta_{\text{p}}^{2}=0.03,$ did not reach significance. Compared to control participants, in depressed individuals, negative and neutral stimuli induced significantly stronger implicit negative affect, *t*s(62) ≥ 2.81, *p*s ≤ 0.007, *d*s ≥ 0.71, whereas the two groups did not differ regarding positive stimuli, *t*(62) = 1.40, *p* = 0.168, *d* = 0.35. Means of the affective ratings after verbal processing, mental imagery and looking at pictures for the depressed and control groups are illustrated in Figures 2–4.

### Association between the SUIS and positive imagery in depression

**Figure 5:**
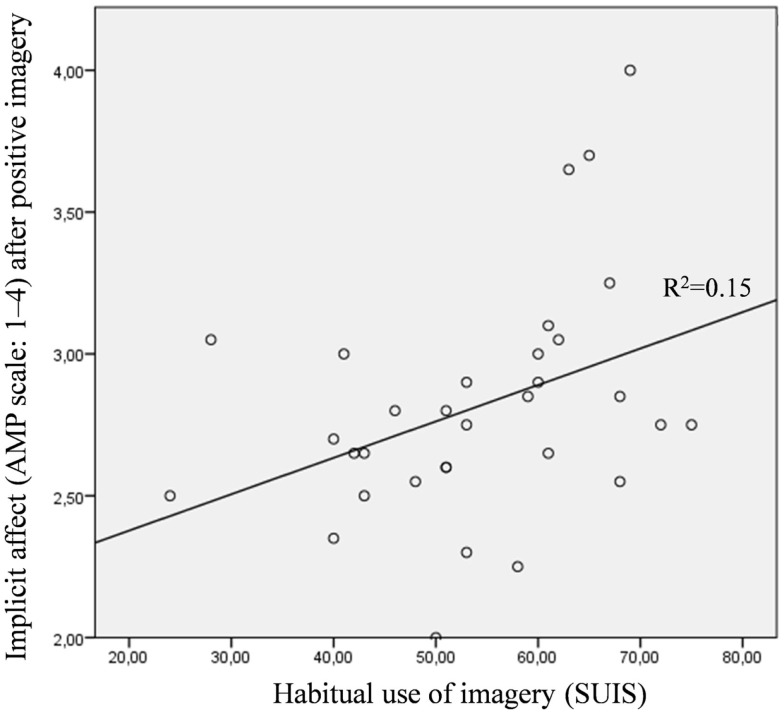
**Scatter plot for the depressed group with the habitual use of mental imagery assessed with the spontaneous use of imagery scale (SUIS) on the *x*-axis and the implicit positive affectivity (AMP, scale: 1–4) after positive mental imagery on the *y*-axis**.

**Figure 6:**
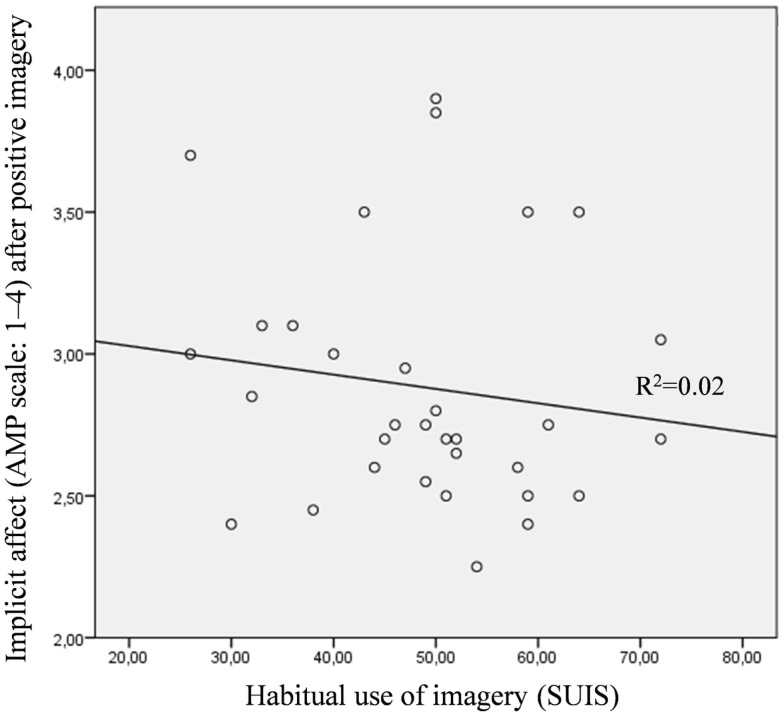
**Scatter plot for the control group with the habitual use of mental imagery assessed with the spontaneous use of imagery scale (SUIS) on the *x*-axis and the implicit positive affectivity (AMP, scale: 1–4) after positive mental imagery on the *y*-axis**.

Among people with a depressive disorder, the everyday use of mental imagery (SUIS) was significantly positively correlated with implicitly assessed affect after positive imagery (*r* = 0.38, *p* < 0.05), but not with explicitly assessed positive affect after positive imagery (*r* = 0.21, *p* = 0.256; hypothesis 4). In the control group, both relationships did not reach significance (implicit: *r* = −0.14, *p* = 0.45; explicit: *r* = 0.32, *p* = 0.07). The relationships between the SUIS and implicitly assessed positive affect after positive mental imagery for the depressed and control groups are illustrated in Figures 5 and 6.

## Discussion

Mental imagery is proposed to play an important role in the amplification and maintenance of depressed mood (2). Few studies, however, have examined this link in clinical depression. The primary aim of the present study was to examine the affective consequences of mental imagery using an implicit as well as an explicit measure. By comparing the emotional impact of mental images, verbally processed stimuli, and “real” pictures, we could also determine the specific impact of mental images.

In contrast to the control group, individuals with a depressive disorder did not exhibit an emotion-amplifying effect of mental imagery compared to verbal processing. Pictures induced the strongest explicitly assessed affect in depression. Due to the missing emotion-amplifying effect of mental imagery in depression compared to the other processing modalities, we could not confirm that mental images are “particularly toxic” in depression [Ref. (2) p. 24]. Compared with the explicit measure, the ANOVA for the implicit measure showed no significant three-way interaction of group by condition by valence. This finding might be attributed to generally smaller effects in the implicit measure and consequent difficulties in detecting significant results in the overall analysis.

There are several possible explanations for the findings that mental imagery had no emotion-amplifying effect compared to verbal processing and that pictures were particularly emotion-amplifying in depression (specifically for depression in the explicit measure). To generate mental images (e.g., of “vacation”), memories are retrieved from long-term memory (29) and working memory then plays a crucial role in maintaining and modifying these mental images (48). Previous work has related negative affect and depression to impaired working memory performance, a finding that could be explained by two assumptions (49, 50): negative feelings and emotion-regulation processes require cognitive resources [resource allocation model (51)] and/or depression may be associated with deficits in the use of spontaneous response strategies (52). Individuals with depressive symptoms clearly benefited from directed strategies regarding their performance in a memory task (52). In addition, previous studies have found that depressed individuals report significantly fewer specific memories (53). Generic memories and images may result in less intense emotional reactions (53, 54). Indeed, in this study, depressed individuals rated the generation of mental images as more difficult or demanding than did control participants. The retrieval of generic memories may also represent an emotion–regulation or avoidance mechanism, particularly to avoid further mood deterioration (53, 55). In addition, verbal processing may trigger a ruminative-thinking style in depression (56, 57), which reinforces negative affect and may have an emotional impact comparable to that of mental imagery.

An additional explanation for the reduced emotional impact of mental imagery in depression, especially for the less efficient positive imagery processes, might be that mental images trigger increased self-focus and comparisons with a more positive past or happier people. This discrepancy might hinder mood improvement or the mood improvement might be very transient (58). Indeed, our findings support this assumption. In depressed participants, positive mental images elicited significantly stronger implicitly assessed positive affect than did positive pictures. Additionally, in the implicit measure, the benefit of positive imagery was comparable in the two groups, indicating that depressed individuals may benefit from positive imagery on an automatic or implicit level.

Interestingly, increases in negative affect in depression compared to healthy controls were observed in both the explicit and implicit measure. In contrast, decreased positive affect in depression was only found in the explicit measure. These findings suggest that deficits in the processing of positive material, which were observed in depression [e.g., Ref. (59, 60)] may be related to decreased elaboration and shallow levels of processing during confronting with positive material. In this regard, our study also extends previous studies on implicit processes in depression, because most studies have focused on memory and attention [e.g., Ref. (59)] and only little research has concentrated on the emotional impact on mood and hedonic perception, aspects which are crucial in the context of depression.

Concerning the habitual use of mental imagery, we found a positive correlation between the everyday use of mental imagery and implicit positive affect after positive imagery in patients with depression. It is possible that practice could promote the benefit of positive imagery in depression or that depressed individuals who show more positive affect after positive imagery make an attempt to harness this in everyday life. Due to the correlational nature of this finding, we cannot draw any causal conclusions at this stage. However, overall, our results concerning positive imagery suggest both that the promotion of pleasant imagery may be a promising treatment intervention and that there is potential in depressed people to benefit from positive imagery (2, 34). Using an imagery-based cognitive interpretation modification (CBM) paradigm, Lang et al. (34) found that depressed individuals who had practiced positive imagery showed a significant reduction of intrusions and depressive symptoms from pre- to post-treatment. Our results also suggest that such interventions might benefit from practicing the vividness of positive imagery and from frequent everyday use of vivid positive imagery. For example, Werner-Seidler and Moulds (61) recently demonstrated that depressed individuals who practice a concrete processing style benefit from the retrieval of positive memories.

Furthermore, the overall lower emotional responses in the depressed group could reflect a differently calibrated neutral point in hedonic detection in depression. According to Baddeley (62), depression could be characterized by an inappropriate high-neutral point in the hedonic detection system, which is related to a pessimistic outlook and negative responses. Consistent with the preponderance of mood-congruent memories in depression (63), the heightened neutral point could be associated with a preferential retrieval of negative information, resulting in the reinforcement of depressed mood (62). These lower affective reactions in depression, respectively, the differences between both groups were larger regarding negative and neutral stimuli than positive stimuli. This finding is in accordance with theoretical models of depression [e.g., Beck’s cognitive theory, Ref. (3)] and previous studies suggesting that depression is associated with a deeper processing of negative stimuli, deficits in controlling and disengaging from negative information, and a negative interpretation bias of ambiguous stimuli (55, 64).

### Study limitations and future research

The mechanism that is responsible for the AMP effect is currently being discussed (32, 65–67). Some authors assume that the AMP effect can be better explained as a semantic misattribution than as an affective misattribution (66). In contrast, Gawronski and Ye (32) demonstrated that the AMP is able to assess implicit affect as well. Regarding the role of intention and awareness, the psychometric quality of the AMP was higher when participants reported on intentional ratings of the prime stimuli instead of the Chinese characters (65). Payne et al. (67) criticized the conclusions by Bar-Anan and Nosek (65) by arguing, for example, that self-reports are less appropriate for assessing the cause of participants’ reactions.

A previous study found that the latency to generate mental images was significantly longer for individuals with depressed mood compared to control participants (68). Although our results show no differences between the depressed and control group regarding the reported ability to generate mental images and to concentrate on mental images for the entire time period (see Table 1), we cannot rule out the possibility that the time duration for generating mental images might have been too short for people with depression. The comparatively rapid sequence of the AMP requires relatively short prime stimuli. So, the time of 3 s was a tradeoff between brevity (in order to assess automatic and spontaneous affective priming effects in the AMP) and the minimum of time necessary to generate a mental image.

In the verbal condition used by Holmes and colleagues (10, 69), participants were asked to focus on the meaning of the presented descriptions, which lasted about 10–13 s. In contrast, the comparatively rapid sequence of events in the AMP requires relatively short prime stimuli. While focusing on the meaning of only single words (compared to more complex scenes) for 3 s, it could be that mental images spontaneously pop up in the mind. So, counting syllables seemed very promising in the context of the AMP. However, the counting syllables task requires rather shallow processing and imposed rather low cognitive load. So, we cannot rule out that these differences between the tasks influenced the results.

In the picture condition, participants were generally instructed to look at and concentrate on the presented pictures. Besides the questions concerning ability, concentration, and difficulty, we have no more information in which manner participants conducted this task (e.g., focusing on their meaning or on picture characteristics). So, it would have been interesting to investigate if people (e.g., with higher depression scores) process pictures differently (that might have an influence on our results).

A relatively low number of trials were presented in the implicit as well as explicit measure: 20 trials per condition and valence. In addition, due to the overall analyses with three factors and multiple levels [2 (group: DG vs. CG) × 3 (condition: mental imagery vs. verbal processing vs. pictures) × 3 (valence: negative vs. neutral vs. positive)] the study might be limited to detect (more) clear differences between the groups and conditions.

Furthermore, some previous studies varied the observer perspective of the created mental images (12, 69). Holmes et al. (69) found that compared to an observer perspective, the field perspective (seeing with one’s own eyes) caused more positive affect after positive mental imagery. In future studies, it would be very interesting to systematically investigate the effects of the observer perspective of negative and positive mental imagery in clinical depression. Given an emotional dampening effect of the observer perspective, it could be beneficial if people with depression use an observer perspective for negative events, but a field perspective for positive events (69).

Future research should test our findings using other experimental tasks to rule out an effect of the specific method. Specifically, the result that depressed and healthy control individuals experienced comparable implicit affect after positive imagery should be tested with an alternative implicit method. A further issue worth studying is whether depressed individuals suppress (concrete) negative mental images, reflecting an avoidance mechanism in depression comparable to the avoidance theory of worry in the context of generalized anxiety disorder (70). Additionally, the use of imagery techniques seems to present a further promising treatment intervention for depression (34), which would be worthwhile to investigate further and to improve (for example, by promoting vividness).

In sum, evidence for an emotion-amplifying effect of mental imagery compared to verbal processing was only found in the control group, but not in individuals with depression. Thus, the especially “toxic” effect of mental imagery compared to other processing modalities in depression could not be confirmed. On an implicit or automatic level, depressed individuals benefited from positive imagery comparable to control participants. This benefit was found particularly in depressed individuals with frequent everyday use of mental imagery. Our results suggest that cognitive–behavioral interventions in the treatment of depression, which often include the modification of negative verbal cognitions, might benefit from additional imagery techniques, particularly the promotion of positive imagery (2, 34).

## Author Contributions

Conception and design of the work: SG, MW; acquisition: SG; analysis: SG; interpretation of data: all authors (SG, JJ, WH, MW). Writing the article: SG, Critical revision of the article: JJ, WH, MW. Final approval of the version to be published: all authors (SG, JJ, WH, MW). Agreement to be accountable for all aspects of the work in ensuring that questions related to the accuracy or integrity of any part of the work are appropriately investigated and resolved: all authors (SG, JJ, WH, MW).

## Conflict of Interest Statement

The authors declare that the research was conducted in the absence of any commercial or financial relationships that could be construed as a potential conflict of interest.

## Supplementary Material

The Supplementary Material for this article can be found online at http://journal.frontiersin.org/article/10.3389/fpsyt.2015.00094

## References

[1] Kosslyn SM Ganis G Thompson WL Neural foundations of imagery. Nat Rev Neurosci (2001) 2(9):635–42. 10.1038/35090055 11533731

[2] Holmes EA Lang TJ Deeprose C. Mental imagery and emotion in treatment across disorders: using the example of depression. Cogn Behav Ther (2009) 38:21–8. 10.1080/16506070902980729 19697177

[3] Beck AT Cognitive Therapy and the Emotional Disorders. New York, NY: International University Press (1976).

[4] Birrer E Michael T Munsch S. Intrusive images in PTSD and in traumatised and non-traumatised depressed patients: a cross-sectional clinical study. Behav Res Ther (2007) 45(9):2053–65. 10.1016/j.brat.2007.03.005 17481577

[5] Patel T Brewin CR Wheatley J Wells A Fisher P Myers S. Intrusive images and memories in major depression. Behav Res Ther (2007) 45(11):2573–80. 10.1016/j.brat.2007.06.004 17669359

[6] Reynolds M Brewin CR. Intrusive memories in depression and posttraumatic stress disorder. Behav Res Ther (1999) 37(3):201–15. 10.1016/S0005-7967(98)00132-6 10087639

[7] Holmes EA Lang TJ Moulds ML Steele AM. Prospective and positive mental imagery deficits in dysphoria. Behav Res Ther (2008) 46(8):976–81. 10.1016/j.brat.2008.04.009 18538304

[8] Deeprose C Holmes EA. An exploration of prospective imagery: the impact of future events scale. Behav Cogn Psychother (2010) 38:201–9. 10.1017/S1352465809990671 20074386

[9] Morina N Deeprose C Pusowski C Schmid M Holmes EA. Prospective mental imagery in patients with major depressive disorder or anxiety disorders. J Anxiety Disord (2011) 25:1032–7. 10.1016/j.janxdis.2011.06.012 21783339 PMC3389342

[10] Holmes EA Mathews A Mental imagery and emotion: a special relationship? Emotion (2005) 5(4):489–97. 10.1037/1528-3542.5.4.489 16366752

[11] Holmes EA Mathews A Mackintosh B Dalgleish T. The causal effect of mental imagery on emotion assessed using picture-word cues. Emotion (2008) 8(3):395–409. 10.1037/1528-3542.8.3.395 18540755

[12] Nelis S Vanbrabant K Holmes EA Raes F Greater positive affect change after mental imagery than verbal thinking in a student sample. J Exp Psychopathol (2012) 3(2):178–88. 10.5127/jep.021111 PMC4599135 26457173

[13] Holmes EA Mathews A. Mental imagery in emotion and emotional disorders. Clin Psychol Rev (2010) 30(3):349–62. 10.1016/j.cpr.2010.01.001 20116915

[14] Blackwell SE Holmes EA Modifying interpretation and imagination in clinical depression: a single case series using cognitive bias modification. Appl Cogn Psychol (2010) 24:338–50. 10.1002/acp.1680

[15] Ceschi G Banse R van der Linden M Implicit but stable. Mental imagery changes explicit but not implicit anxiety. Swiss J Psychol (2009) 68(4):213–20. 10.1024/1421-0185.68.4.213

[16] Görgen SM Joormann J Hiller W Witthöft M Implicit affect after mental imagery: introduction of a novel measure and relations to depressive symptoms in a non-clinical sample. J Exp Psychopathol (2015) 6(1):59–81. 10.5127/jep.041114

[17] Watson D Clark LA Tellegen A Development and validation of brief measures of positive and negative affect: the PANAS scales. J Pers Soc Psychol (1988) 54(6):1063–70. 10.1037/0022-3514.54.6.1063 3397865

[18] Egloff B Schmukle SC. Predictive validity of an implicit association test for assessing anxiety. J Pers Soc Psychol (2002) 83(6):1441–55. 10.1037//0022-3514.83.6.1441 12500823

[19] Quirin M Kázen M Rohrmann S Kuhl J. Implicit but not explicit affectivity predicts circadian and reactive cortisol: using the implicit positive and negative affect test. J Pers (2009) 77(2):401–25. 10.1111/j.1467-6494.2008.00552.x 19192075

[20] Robinson MD Clore GL. Belief and feeling: evidence for an accessibility model of emotional self-report. Psychol Bull (2002) 128(6):934–60. 10.1037//0033-2909.128.6.934 12405138

[21] Teachman BA Joormann J Steinman SA Gotlib IH. Automaticity in anxiety disorders and major depressive disorder. Clin Psychol Rev (2012) 32(6):575–603. 10.1016/j.cpr.2012.06.004 22858684 PMC3419810

[22] Beevers CG. Cognitive vulnerability to depression: a dual process model. Clin Psychol Rev (2005) 25(7):975–1002. 10.1016/j.cpr.2005.03.003 15905008

[23] Haeffel GJ Abramson LY Brazy PC Shah JY Teachman BA Nosek BA. Explicit and implicit cognition: a preliminary test of a dual-process theory of cognitive vulnerability to depression. Behav Res Ther (2007) 45(6):1155–67. 10.1016/j.brat.2006.09.003 17055450

[24] Phillips WJ Hine DW Thorsteinsson EB. Implicit cognition and depression: a meta-analysis. Clin Psychol Rev (2010) 30(6):691–709. 10.1016/j.cpr.2010.05.002 20538393

[25] Greenwald AG McGhee DE Schwartz JLK. Measuring individual differences in implicit cognition: the implicit association test. J Pers Soc Psychol (1998) 74(6):1464–80. 10.1037/0022-3514.74.6.1464 9654756

[26] De Houwer J The extrinsic affective Simon task. Exp Psychol (2003) 50(2):77–85. 10.1027//1618-3169.50.2.77 12693192

[27] Payne BK Cheng CM Govorun O Stewart BD. An inkblot for attitudes: affect misattribution as implicit measurement. J Pers Soc Psychol (2005) 89(3):277–93. 10.1037/0022-3514.89.3.277 16248714

[28] Lang PJ A bio-informational theory of emotional imagery. Psychophysiology (1979) 16(6):495–512. 10.1111/j.1469-8986.1979.tb01511.x 515293

[29] Ganis G Thompson WL Kosslyn SM. Brain areas underlying visual mental imagery and visual perception: an fMRI study. Brain Res Cogn Brain Res (2004) 20(2):226–41. 10.1016/j.cogbrainres.2004.02.012 15183394

[30] Hirsch CR Meynen T Clark DM. Negative self-imagery in social anxiety contaminates social interactions. Memory (2004) 12(4):496–506. 10.1080/09658210444000106 15487545

[31] Pictet A Coughtrey AE Mathews A Holmes EA. Fishing for happiness: the effects of generating positive imagery on mood and behaviour. Behav Res Ther (2011) 49:885–91. 10.1016/j.brat.2011.10.003 22032936 PMC3240747

[32] Gawronski B Ye Y. What drives priming effects in the affect misattribution procedure? Pers Soc Psychol Bull (2014) 40(1):3–15. 10.1177/0146167213502548 23982152

[33] Payne BK Burkley MA Stokes MB. Why do implicit and explicit attitude tests diverge? The role of structural fit. J Pers Soc Psychol (2008) 94(1):16–31. 10.1037/0022-3514.94.1.16 18179315

[34] Lang TJ Blackwell SE Harmer CJ Davison P Holmes EA. Cognitive bias modification using mental imagery for depression: developing a novel computerized intervention to change negative thinking styles. Eur J Pers (2012) 26(2):145–57. 10.1002/per.855 23316101 PMC3532611

[35] Williams AD Blackwell SE Mackenzie A Holmes EA Andrews G. Combining imagination and reason in the treatment of depression: a randomized controlled trial of internet-based cognitive-bias modification and internet-CBT for depression. J Consult Clin Psychol (2013) 81(5):793–9. 10.1037/a0033247 23750459 PMC3780629

[36] Reisberg D Pearson DG Kosslyn SM Intuitions and introspections about imagery: the role of imagery experience in shaping an investigator’s theoretical views. Appl Cogn Psychol (2003) 17(2):147–60. 10.1002/acp.858

[37] Wittchen HU Zaudig M Fydrich T Strukturiertes Klinisches Interview für DSM-IV, Achse I und II (SKID) [Structured Clinical Interview for DSM-IV Axis I and II Disorders (SCID)]. Göttingen: Hogrefe (1997).

[38] Association AP Diagnostic and Statistical Manual of Mental Disorders (4th ed., Text Revision). Washington, DC: Association AP (2000).

[39] Bradley MM Lang PJ. Measuring emotion: the self-assessment manikin and the semantic differential. J Behav Ther Exp Psychiatry (1994) 25(1):49–59. 10.1016/0005-7916(94)90063-9 7962581

[40] Võ MLH Conrad M Kuchinke L Urton K Hofmann MJ Jacobs AM The Berlin affective word list reloaded (BAWL-R). Behav Res Methods (2009) 41(2):534–8. 10.3758/BRM.41.2.534 19363195

[41] Võ MLH Jacobs AM Conrad M. Cross-validating the Berlin affective word list. Behav Res Methods (2006) 38(4):606–9. 10.3758/BF03193892 17393831

[42] Lang PJ Bradley MM Cuthbert BN International Affective Picture System (IAPS): Affective Ratings of Pictures and Instruction Manual. Technical Report A-8. Gainesville, FL: University of Florida (2008).

[43] Beck AT Steer RA Brown GK Manual for the Beck Depression Inventory-II. San Antonio, TX: Psychological Corporation (1996).

[44] Hautzinger M Keller F Kühner C Beck Depressions-Inventar. 2. Auflage (BDI-II). [Beck Depression-Inventory. 2. Edition]. Frankfurt: Harcourt Test Services (2006).

[45] Kühner C Bürger C Keller F Hautzinger M. Reliabilität und Validität des revidierten Beck-Depressionsinventars (BDI-II) [Reliability and validity of the revised German version of the Beck depression inventory]. Nervenarzt (2007) 78(6):651–6. 10.1007/s00115-006-2098-7 16832698

[46] Görgen SM Hiller W Witthöft M Die spontaneous use of imagery scale (SUIS). Entwicklung und teststatistische Prüfung einer deutschen Adaption [The spontaneous use of imagery scale (SUIS). development and psychometric evaluation of a German adaptation]. Diagnostica (in press).

[47] Cohen J Statistical Power Analysis for the Behavioral Sciences. New York, NY: Erlbaum (1988).

[48] Baddeley AD Andrade J Working memory and the vividness of imagery. J Exp Psychol Gen (2000) 129(1):126–45. 10.1037/0096-3445.129.1.126 10756490

[49] Brose A Schmiedek F Lövdén M Lindenberger U. Daily variability in working memory is coupled with negative affect: the role of attention and motivation. Emotion (2012) 12(3):605–17. 10.1037/a0024436 21787075

[50] Klimkeit EI Tonge B Bradshaw JL Melvin GA Gould K Neuropsychological deficits in adolescent unipolar depression. Arch Clin Neuropsychol (2011) 26(7):662–76. 10.1093/arclin/acr051 21690097

[51] Ellis HC Ashbrook PW Resource allocation model of the effects of depressed mood states on memory. In: Fiedler K Forgas J, editors. Affect, Cognition, and Social Behavior. Göttingen: Hogrefe (1988). p. 25–43.

[52] Hertel PT Hardin TS. Remembering with and without awareness in a depressed mood: evidence of deficits in initiative. J Exp Psychol Gen (1990) 119(1):45–59. 10.1037/0096-3445.119.1.45 2141063

[53] Williams JMG Barnhofer T Crane C Herman D Raes F Watkins E Autobiographical memory specificity and emotional disorder. Psychol Bull (2007) 133(1):122–48. 10.1037/0033-2909.133.1.122 17201573 PMC2834574

[54] Williams JMG Stiles WB Shapiro DA Cognitive mechanisms in the avoidance of painful and dangerous thoughts: elaborating the assimilation model. Cogn Ther Res (1999) 23(3):285–306. 10.1023/A:1018743615228

[55] Gotlib IH Joormann J Cognition and depression: current status and future directions. Annu Rev Clin Psychol (2010) 6(1):285–312. 10.1146/annurev.clinpsy.121208.131305 20192795 PMC2845726

[56] Fresco DM Frankel AN Mennin DS Turk CL Heimberg RG Distinct and overlapping features of rumination and worry: the relationship of cognitive production to negative affective states. Cogn Ther Res (2002) 26(2):179–88. 10.1023/A:1014517718949

[57] Nolen-Hoeksema S Wisco BE Lyubomirsky S Rethinking rumination. Perspect Psychol Sci (2008) 3(5):400–24. 10.1111/j.1745-6924.2008.00088.x 26158958

[58] Joormann J Siemer M. Memory accessibility, mood regulation, and dysphoria: difficulties in repairing sad mood with happy memories? J Abnorm Psychol (2004) 113(2):179–88. 10.1037/0021-843X.113.2.179 15122938

[59] Levens SM Gotlib IH. Updating positive and negative stimuli in working memory in depression. J Exp Psychol Gen (2010) 139(4):654–64. 10.1037/a0020283 21038984 PMC2984552

[60] LeMoult J Yoon KL Joormann J Affective priming in major depressive disorder. Front Integr Neurosci (2012) 6:76 10.3389/fnint.2012.00076 23060758 PMC3464437

[61] Werner-Seidler A Moulds ML. Mood repair and processing mode in depression. Emotion (2012) 12(3):470–8. 10.1037/a0025984 22023367

[62] Baddeley A Working memory and emotion: ruminations on a theory of depression. Rev Gen Psychol (2013) 17(1):20–7. 10.1037/a0030029

[63] Joormann J Gotlib IH. Emotion regulation in depression: relation to cognitive inhibition. Cogn Emot (2010) 24(2):281–98. 10.1080/02699930903407948 20300538 PMC2839199

[64] Hamilton JP Gotlib IH. Neural substrates of increased memory sensitivity for negative stimuli in major depression. Biol Psychiatry (2008) 63(12):1155–62. 10.1016/j.biopsych.2007.12.015 18281017 PMC2474758

[65] Bar-Anan Y Nosek BA. Reporting intentional rating of the primes predicts priming effects in the affective misattribution procedure. Pers Soc Psychol Bull (2012) 38(9):1194–208. 10.1177/0146167212446835 22611055

[66] Blaison C Imhoff R Hühnel I Hess U Banse R. The affect misattribution procedure: hot or not? Emotion (2012) 12(2):403–12. 10.1037/a0026907 22390705

[67] Payne BK Brown-Iannuzzi J Burkley M Arbuckle NL Cooley E Cameron CD Intention invention and the affect misattribution procedure: reply to Bar-Anan and Nosek (2012). Pers Soc Psychol Bull (2013) 39(3):375–86. 10.1177/0146167212475225 23401479

[68] Cocude M Charlot V Denis M Latency and duration of visual mental images in normal and depressed subjects. J Ment Imagery (1997) 21(1–2):127–42.

[69] Holmes EA Coughtrey AE Connor A. Looking at or through rose-tinted glasses? Imagery perspective and positive mood. Emotion (2008) 8(6):875–9. 10.1037/a0013617 19102599

[70] Borkovec TD Alcaine OM Behar E Avoidance theory of worry and generalized anxiety disorder. In: Heimberg RG Turk CL Mennin DS, editors. Generalized Anxiety Disorder: Advances in Research and Practice. New York, NY: Guilford (2004). p. 77–108.

