# Supplementary material for: The Role of Mental Imagery in Depression: Negative Mental Imagery Induces Strong Implicit and Explicit Affect in Depression
Source: Front Psychiatry. 2015 Jul 7;6:94. doi: 10.3389/fpsyt.2015.00094 (PMC4493655; doi:10.3389/fpsyt.2015.00094)
Supplement: Supplementary file 1 [file data_sheet_1.pdf]

Appendix

Word stimuli from the Berlin Affective Word List Reloaded (BAWL-R) and picture stimuli from the International Affective Picture System (IAPS; slide number)

	Mental Imagery		Verbal Processing		Pictures	
**valence**	**implicit**	**explicit**	**implicit**	**explicit**	**implicit**	**explicit**
negative	bomb	alarm clocks	bill	dead	2095	2053
	cemetery	bacterium	carrion	decay	2301	2205
	cockroach	coffin	contusion	fat	2688	2683
	devil	crutch	corpse	moan	3301	2710
	grave	defendant	faint	muck	6834	2981
	hatchet	dungeon	moth	munition	9250	3103
	illness	escape	murder	nausea	9423	6821
	prison	gurney	muzzle	quarrel	9429	8485
	mosquito	thief	soldier	rival	9611	9184
	waste	weapon	wart	urn	9940	9620
neutral	battery	barrel	boot	aquarium	2191	2272
	belt	bottle	carpet	carton	2377	2308
	document	broom	fishing rod	comb	2575	2441
	excavator	cupboard	flag	drum	2635	2485
	folder	curve	lecture	finger	5534	2506
	glass	microphone	pot	glue	7001	7019
	nose	screw	sponge	hammer	7057	7021
	scissors	shirt	spoon	heel	7058	7037
	stairs	stamp	stone	stem	7287	7513
	vest	suitcase	wall*	tray	7595	8325
positive	birthday	admirer	birth	chocolate	2152	1710
	celebration	applause	chick	ecstasy	2340	2045
	eroticism	cash	cheers	fun	2345	2208
	flirt	hero	cookie	journey	4597	2347
	gift	medal	euphoria	luxury	5210	2550
	hit	palace	joy	passion	5621	4599
	ice cream	party	ocean	pearl	5623	4659
	cake	rabbit	sport	sapphire	5833	5781
	vacation	sun	strawberry	shower	8370	5825
	winner	victory	treasure	spring	8380	8492

* The used word “Mauer” in German is unambiguous (e.g., the Berlin wall), in contrast to the English translation “wall” which can have different meanings.
